# Baicalein Protects against Type 2 Diabetes via Promoting Islet *β*-Cell Function in Obese Diabetic Mice

**DOI:** 10.1155/2014/846742

**Published:** 2014-07-23

**Authors:** Yu Fu, Jing Luo, Zhenquan Jia, Wei Zhen, Kequan Zhou, Elizabeth Gilbert, Dongmin Liu

**Affiliations:** ^1^Department of Human Nutrition, Foods & Exercises, College of Agriculture and Life Sciences, Virginia Tech, 1981 Kraft Drive, Corporate Research Center, Blacksburg, VA 24061, USA; ^2^Department of Biology, The University of North Carolina at Greensboro, Greensboro, NC 27412, USA; ^3^Department of Nutrition and Food Science, Wayne State University, Detroit , MI 48202, USA

## Abstract

In both type 1 (T1D) and type 2 diabetes (T2D), the deterioration of glycemic control over time is primarily caused by an inadequate mass and progressive dysfunction of *β*-cell, leading to the impaired insulin secretion. Here, we show that dietary supplementation of baicalein, a flavone isolated from the roots of Chinese herb *Scutellaria baicalensis*, improved glucose tolerance and enhanced glucose-stimulated insulin secretion (GSIS) in high-fat diet (HFD-) induced middle-aged obese mice. Baicalein had no effect on food intake, body weight gain, circulating lipid profile, and insulin sensitivity in obese mice. Using another mouse model of type 2 diabetes generated by high-fat diet (HFD) feeding and low doses of streptozotocin injection, we found that baicalein treatment significantly improved hyperglycemia, glucose tolerance, and blood insulin levels in these middle-aged obese diabetic mice, which are associated with the improved islet *β*-cell survival and mass. In the *in vitro* studies, baicalein significantly augmented GSIS and promoted viability of insulin-secreting cells and human islets cultured either in the basal medium or under chronic hyperlipidemic condition. These results demonstrate that baicalein may be a naturally occurring antidiabetic agent by directly modulating pancreatic *β*-cell function.

## 1. Introduction

Diabetes mellitus is a growing public health concern in the United States, presently affecting 25.8 million or 8.3% of the American population [1]. This number is still increasing and is predicted to double by 2025 [2]. The medical costs associated with this disease, which include drug therapy and surgical treatment, are tremendous [1]. Diabetes is a complex metabolic disorder characterized by abnormalities in insulin secretion and action, which leads to hyperglycemia [3]. Type 1 diabetes (T1D) is a T-cell-mediated autoimmune disease leading to the destruction of pancreatic *β*-cells, whereas type 2 diabetes (T2D) is due to a combination of peripheral insulin resistance and loss of functional *β*-cell mass [4–7]. Therefore, in both T1D and T2D, inadequate *β*-cell mass and *β*-cell dysfunction leading to impaired insulin secretion are central to the deterioration of glycemic control [8]. Thus, the search for novel and cost-effective agents that can enhance *β*-cell function and preserve *β*-cell mass is important to provide effective treatment for diabetes.

*Scutellariae baicalensis Georgi *(SBG), a medicinal plant, grows in some Asian countries including Siberia, far East of Russia, Mongolia, China, and some other Eastern Asian countries [9]. The root of SBG has been used as an ingredient in traditional Chinese medicine formulations for thousands of years [10]. According to Chinese medicine, SBG has various beneficial effects on health. There have been 295 compounds isolated from SBG, more than 40 of which are flavonoids [11, 12]. Baicalein, baicalin, wogonin, and wogonoside are presumably the main bioactive components in SBG, which account for 5.41%, 10.11%, 1.3%, and 3.55%, respectively, of the dry material [13]. Recently, baicalein and its glucuronide baicalin have drawn wide attention due to their possible health effects. A recent study showed that oral administration of baicalin for 30 days reduced hyperglycemia-induced mitochondrial damage in *β*-cells in diabetic rats [14]. Another study reported that long-term administration of baicalin reduced high-fat diet- (HFD-) induced body weight gain, circulating cholesterol and free fatty acid levels, lipid deposits in the liver, and systemic inflammation markers [15]. Studies on whether baicalein has an antidiabetic effect are scarce. Baicalein was reported to have beneficial effects in diabetes-associated health complications [14–17], cancers [18, 19], cardiovascular disease [20], inflammation [21], bacterial infections [22, 23], and oxidative stress [24–26], although some of the claimed benefits remain controversial and the mechanisms of these effects are unclear. In addition, it is unknown whether baicalein has an antidiabetic effect. Moreover, whether this compound exerts a beneficial effect on pancreatic *β*-cells is unclear.

As aforementioned, loss of functional *β*-cell mass and *β*-cell dysfunction is central to the development of diabetes [8]. Thus, agents that can increase *β*-cell mass and/or enhance *β*-cell function can provide effective treatment for diabetes and thereby decrease the burden of morbidity from diabetes and related complications. In the present study, we evaluated the antidiabetic potential of baicalein in two mouse models and further determined its effects on pancreatic *β*-cells. We found for the first time that baicalein improves hyperglycemia and glucose tolerance, promotes insulin secretion, and preserves islet mass by inhibiting apoptosis in middle-aged obese diabetic mice.

## 2. Materials and Methods

### 2.1. Materials

RPMI-1640 media (RPMI) were purchased from Sigma-Aldrich (St. Louis, MO); CMRL-1066 media (CMRL) were purchased from Mediatech (Holly Hill, FL); heat-inactivated fetal bovine serum (FBS) was obtained from HyClone (Logan, UT). Baicalein (98% pure) for* in vitro* studies was purchased from Sigma-Aldrich (St Louis, MO). Stock solutions of baicalein at 20 mM were dissolved in sterilized dimethyl sulfoxide (DMSO) and stored at −80°C before use. Baicalein (98% pure by HPLC) for* in vivo* studies was purchased from Xi'An Yile Bio-Tech Company, China; ultrasensitive rat insulin enzyme-linked immunosorbent assay (ELISA) kits were obtained from Mercodia (Winston-Salem, NC); the active form of the caspase-3 antibody was from BD Biosciences (San Jose, CA); the rabbit polyclonal anti-insulin antibody was from Abcam (Cambridge, MA); the ImmPRESS Anti-rabbit Ig (peroxidase) Polymer Detection kit, Vector NovaRED peroxidase substrate kit, and Vector SG peroxidase substrate kits were from Vector laboratories (Burlingame, CA); cell viability assay kits were from Promega (Madison, WI); and the BrdU ELISA kit for the cell proliferation assay was from Roche Applied Sciences (Indianapolis, IN). All other chemicals were from Sigma-Aldrich. Glucose was dissolved in sterile water and stored at −80°C.

### 2.2. Animals

Eight-month-old male C57BL/6 mice (National Cancer Institute, Frederick, MD) were individually housed in an animal room maintained on a 12 h light/dark cycle under constant temperature (22–25°C) with* ad libitum* access to food and water. After 1 wk of environment acclimation, the following two animal studies were performed. The animal study protocols were reviewed and approved by the Institutional Animal Care and Use Committee at Virginia Tech.

### 2.3. High-Fat Diet-Induced Obese Mice

For the first animal study, mice were divided into 3 groups (*n* = 10) and fed either a standard diet (SD) with 10% of calories derived from fat, a high-fat diet (HF; Research Diets Inc., New Brunswick, NJ) with 58% of calories from fat, or HF supplemented with baicalein (0.5 g/kg diet) for 8 wks. Body weight (BW) and food intake were recorded weekly throughout the study. The fasting blood glucose levels in tail vein blood samples were measured using a glucometer (Roche) every 4 wk. After 7 wk of dietary baicalein supplementation, body composition was evaluated using an LF-90 instrument (Bruker Optics, Inc., Billerica MA). The LF-90 body composition instrument is based on time domain nuclear magnetic resonance (TD-NMR) technology which provides an* in vivo* measurement of lean tissue, body fat, and body fluid in live mice without anesthesia. At the end of 8 wk of dietary treatment, glucose tolerance and insulin tolerance tests were performed. For glucose tolerance tests, mice were fasted for 12 h and injected intraperitoneally (ip) with a single bolus of glucose (l g/kg BW). Glucose levels were measured at time points of 0, 15, 30, 60, and 120 min, and plasma insulin concentrations were measured at 0 and 30 min, after glucose administration. For the insulin tolerance test, mice were injected i.p. with insulin (0.75 units/kg BW), and blood glucose levels were measured at 0, 15, 30, 60, and 120 min after insulin administration. Area under the curve (AUC) was calculated using the trapezoidal rule. At the end of the study, blood samples were collected from overnight-fasted mice; plasma insulin concentration was measured using an ultrasensitive mouse/rat insulin ELISA kit; fasting plasma total cholesterol and triacylglycerols were measured in triplicate by enzymatic methods using a Pointer 180 Analyzer (Pointe Scientific, Canton, MI) as described [27].

### 2.4. Streptozotocin- (STZ-) Induced Diabetic Mice

For this study, mice were divided into 6 groups (*n* = 10 mice/group) with initial fasting blood glucose and body weights balanced among groups. Mice were then fed a SD diet, a HF diet (58 kcal% fat), or HF diet containing 0.25 g or 0.5 g baicalein/kg diet. After 4 wk of treatment, mice received ip injection of STZ dissolved in 0.1 M cold sterile sodium citrate buffer (pH 4.5) at 40 mg/kg daily for 3 consecutive days. Control mice received ip injection of saline. BW and food intake were measured weekly throughout the study. Fasting blood glucose levels were recorded every 2 wk before STZ injection. Following STZ injection, the levels of nonfasting blood glucose were measured weekly to assess the onset of hyperglycemia (nonfasting blood glucose >250 mg/dL) [27]. Plasma insulin concentration measurements and glucose tolerance and insulin tolerance tests were performed as stated above.

### 2.5. Immunohistochemistry

At the end of experiment, mice were euthanized, and the pancreata were dissected and fixed in 4% (vol/vol) formaldehyde buffer (pH 7.2). A series of tissue sections (5 *μ*m thickness) were prepared by AML Laboratory (Baltimore, MD). For determining *β*-cell mass, tissue sections mounted on glass slides were immunofluorescently stained with insulin antibody as we previously described [27]. The *β*-cell area was measured using images acquired from insulin-stained pancreatic sections. The *β*-cell mass was calculated by dividing the area of insulin-positive cells by the total area of pancreatic tissue and multiplying by the pancreas weight. The average was obtained from 10 mice in each group. The double labelling against the activated caspase-3 and insulin as a measure of *β*-cell apoptosis was performed using an ImmPRESS detection system according to the manufacturer's protocol. In brief, tissue sections were deparaffinized in xylene and rehydrated through a graded series of alcohol washes. Endogenous peroxidase activity was blocked by incubating in 3% H_2_O_2_ for 10 min. Antigen retrieval was carried out by boiling sections for 10 min in 0.01 M citrate buffer (pH 6.0), cooled at room temperature for 30 min, and then blocked with 2.5% normal horse serum in PBS for 20 min. Sections were then incubated with a rabbit-anti-mouse caspase-3 antibody detecting the activated form of caspase-3 (1 : 200 dilution) at room temperature for 1 h. Visualization was performed using a NovaRED peroxidase substrate kit. The slides were subsequently processed for double immunostaining for insulin using the same approach, and insulin was visualized using a SG peroxidase substrate kit. Five pancreatic sections from each mouse with 6 mice per group were costained with activated caspase-3 and insulin. Photographs of immune-stained pancreatic sections were captured using an Olympus phase contrast microscope.

### 2.6. Cell and Human Islet Culture

INS832/13 cells (kindly provided by Dr. Christopher Newgard at Duke University) were cultured in RPMI medium containing 11.1 mM glucose and supplemented with 10% FBS, 1 mM sodium pyruvate, 10 mM HEPES, 2 g/L sodium bicarbonate, 50 *μ*M *β*-mercaptoethanol, 100 U/mL penicillin, and 100 *μ*g/mL streptomycin as previously described [27]. Medium was changed every 2-3 days until cells were approximately 70% confluent. Human islets were isolated from cadaver organ donors by the Islet Cell Resource Centers administered by the Southern California Resources Center & Southern California Islet Consortium at the National Medical Center (Duarte, CA). The islet purity was 90–99% and viability was 80–99%. Before the experiment, the islets were maintained in CMRL medium containing 10% FBS.

### 2.7. Insulin Secretion Assay

INS382/13 cells were cultured in 96-well plates in RPMI medium containing 11.1 mM glucose and 10% FBS at 37°C until the cells became 50%–60% confluent. The cultures were then switched to RPMI medium containing 3 mM glucose and 2% FBS for 12 h. For determining the effect of baicalein on glucose-stimulated insulin secretion (GSIS), cells and human islets were washed with Krebs-Ringer bicarbonate (KRB) buffer (129 mM NaCl, 4.8 mM KCl, 1.2 mM MgSO_4_, 1.2 mM KH_2_PO_4_, 2.5 mM CaCl_2_, 5 mM NaHCO_3_, 0.1% BSA, and 10 mM HEPES, pH 7.4) followed by incubation in KRB buffer containing 3 mM or 20 mM glucose in the presence or absence of 0.1, 1, 5, 10, or 20 *μ*M baicalein at 37°C for 30 min. Insulin secreted in supernatants was measured by an ELISA kit. Human islets (100 islets/tube) were incubated in 3 or 20 mM glucose with or without various concentrations of baicalein in a 37°C water bath with gentle shaking for 30 min [28]. Insulin in the supernatants was measured by ELISA and normalized to protein content in the same sample. Our preliminary experiments showed that exposure of the cells to baicalein for 30 min had no effect on insulin or protein content.

### 2.8. Cell Viability Assay

INS382/13 cells or human islets were incubated with various concentrations of baicalein or vehicle in RPMI medium containing 5.5 mM glucose and 2% FBS at 37°C. 48 h later, the cultures were continued for an additional 4 h in the presence of 3-(4,5-dimethylthiazol-2-yl)-5-(3-carboxymethoxyphenyl)-2-(4-sulfophenyl)-2H-tetrazolium (MTS), a formazan compound, which was reduced by cells into a colored formazan product that was soluble in culture medium. Cell viability was assessed by the absorbance measurements at 490 nm with a plate reader. To determine whether baicalein has a protective effect on *β*-cells exposed to chronic hyperlipidemia, INS382/13 cells or human islets (150 islets/well) were cultured in RPMI medium containing 0.5 mM palmitate or vehicle with or without various concentrations of baicalein for 4 d, with the medium replaced every other day. The cell viability was determined as stated above.

### 2.9. Cell Proliferation Assay

INS382/13 cells were incubated with various concentrations of baicalein or vehicle in RPMI medium containing 5.5 mM glucose and 2% FBS at 37°C. 24 h later, the cultures were continued for an additional 4 h in the presence of bromodeoxyuridine (BrdU), an analog of thymidine which incorporates into newly synthesized DNA, thereby labeling replicating cells. Cell proliferation was assessed by BrdU incorporation measurements with an ELISA cell proliferation assay kit as previously described [29].

### 2.10. Statistical Analyses

All data were analyzed with one-way ANOVA or Student's *t*-test using the SigmaPlot (Systat Software) program. Post hoc treatment comparisons were carried out with Tukey's test. A *P* < 0.05 was considered significant.

## 3. Results

### 3.1. Dietary Intake of Baicalein Had No Effects on Food Intake, Body Weight, Body Composition, and Plasma Lipid Profile in HF Diet-Induced Obese Mice

**Figure 1:**
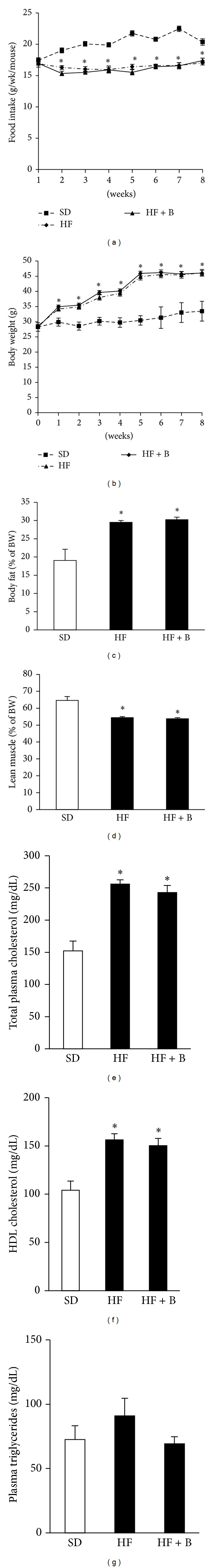
Baicalein supplementation had no effects on food consumption, BW, body composition, or the plasma lipid profile in HF diet-induced obese mice. C57BL/6 male, retired breeder (8 mo) mice were used for this study. Food intake was recorded every 2 or 3 days and cumulative weekly food consumption was calculated (a). BW was monitored every wk (b). Body composition during the final week of the trial was measured with fat tissue mass (c) and lean mass (d) expressed as a percentage of the BW. At the end of the 8th wk of the experiment, fasting plasma total cholesterol (e), HDL-cholesterol (f), and triglycerides (g) were measured. Values represent mean ± SE (*n* = 10/group).  *P < 0.05 versus SD group. BW: body weight, SD: standard diet, HF: high-fat diet, and HF + B: HF + 0.5 g baicalein/kg diet.

The HF diet decreased the accumulative average food intake, but baicalein supplementation for 8 consecutive wk did not alter the food consumption pattern compared with HF diet-fed mice (Figure 1(a)). Four wk of consuming HF diet significantly increased BW of mice. However, dietary intake of baicalein at 0.5 g/kg diet had no effect on HF diet-induced BW gain (Figure 1(b)). Baicalein treatment also had no effects on the weights of the heart, pancreas, spleen, liver, or kidney as compared to weights of HF mice (data not shown). Consistently, baicalein treatment did not alter body composition of obese mice as determined by measuring their relative percentage of fat (Figure 1(c)) and muscle mass (Figure 1(d)). Fasting blood levels of cholesterol (Figure 1(e)) and HDL-cholesterol (Figure 1(f)) were increased in HF diet-fed obese mice, but they were not significantly altered by dietary intake of baicalein. Fasting plasma triglyceride concentrations (Figure 1(g)) were not significantly altered by HF feeding or baicalein treatment.

### 3.2. Dietary Intake of Baicalein Improved Fasting Blood Glucose and Glucose Tolerance without Altering Peripheral Insulin Sensitivity in HF Diet-Induced Obese Mice

**Figure 2:**
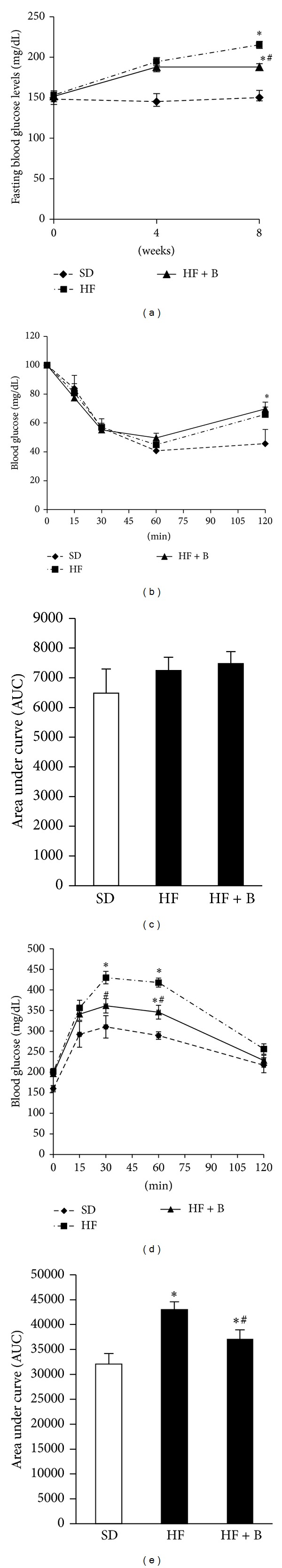
Dietary intake of baicalein improved glucose tolerance but did not affect insulin sensitivity in obese mice. Fasting blood glucose was monitored every 4 wks (a). Insulin tolerance (b) and glucose tolerance tests (d) were performed as stated in the Methods section. The total values of area under the curve (AUC) for insulin (c) and glucose (e) tolerance tests were calculated. Data are means ± SE (*n* = 10 mice/group).  *P < 0.05 versus SD group;  ^#^ *P* < 0.05 versus HF group. SD: standard diet, HF: high-fat diet, and HF + B: HF + 0.5 g baicalein/kg diet.

**Figure 3:**
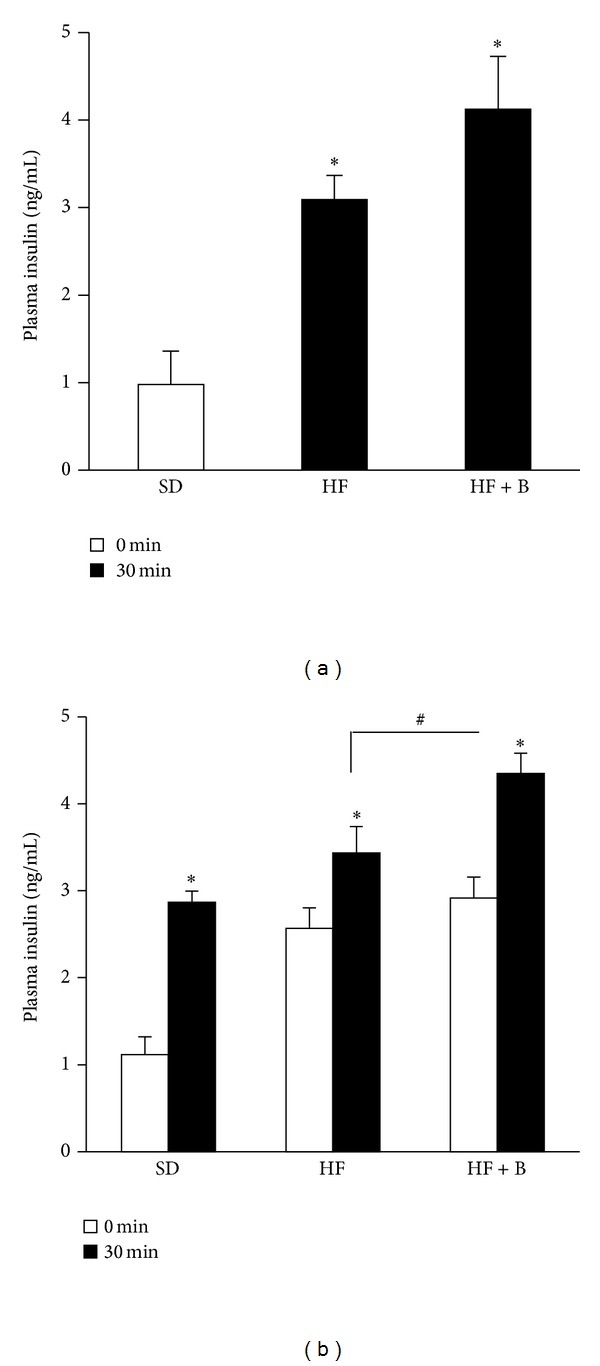
Dietary baicalein intake enhanced glucose-stimulated insulin secretion in obese mice. Fasting plasma insulin concentrations (a) were determined by ELISA after mice were fasted for 12 h. Plasma insulin concentrations at baseline and 30 min postglucose loading via ip injection (1 g/kg BW). Data are means ± SEM (*n* = 10 mice/group).  *P < 0.05 versus SD group;  ^#^ *P* < 0.05 versus HF group. SD: standard diet, HF: high-fat diet, and HF + B: HF + 0.5 g baicalein/kg diet.

After 4 wk of HF diet consumption, mice displayed significantly elevated fasting blood glucose concentrations compared with animals that consumed the SD diet. Baicalein supplementation at this point did not affect the HF diet-induced rise in blood glucose. However, after 8 wks of baicalein supplementation, fasting blood glucose levels were significantly lower (30% reduction) than those in HF-fed mice (Figure 2(a)). Insulin resistance is important to the etiology of T2D and usually occurs in obesity. To determine if dietary intake of baicalein improves insulin sensitivity in obese mice, we performed an intraperitoneal insulin tolerance test. As expected, HF diet treatment impaired peripheral insulin sensitivity. However, there were little differences in plasma glucose levels (Figure 2(b)) and the AUC (Figure 2(c)) postinsulin injection between HF mice and mice given the HF diet supplemented with baicalein. These results suggest that the improvement in glucose tolerance in baicalein-treated mice is likely not due to an improvement in insulin sensitivity. We next performed a glucose tolerance test to evaluate if the improved glycemic control in mice that consumed baicalein-supplemented diets is via protecting pancreatic *β*-cell function. Data showed that baicalein improved glucose tolerance as demonstrated by significantly lower blood glucose levels at 30 and 60 min (Figure 2(d)) as well as reduced AUC (Figure 2(e)) following intraperitoneal glucose injection compared to HF-fed mice. These results suggest that baicalein may promote pancreatic *β*-cell insulin secretory function in response to glucose challenge. We then measured plasma insulin levels after overnight fasting and during intraperitoneal injection of glucose. We observed that fasting plasma insulin levels in HF mice were almost threefold of those in mice that received the SD diet, suggesting that obese mice are insulin resistant (Figure 3(a)). Baicalein treatment had no effect on fasting plasma insulin levels (Figure 3(a)) but significantly increased blood insulin concentrations following glucose injection (Figure 3(b)) indicating that the improvement in glucose tolerance in baicalein-treated mice may be the result of greater insulin secretion from pancreatic *β*-cells.

### 3.3. Dietary Intake of Baicalein Ameliorated Hyperglycemia and Improved Insulin Levels in Middle-Aged Obese Diabetic Mice

**Figure 4:**
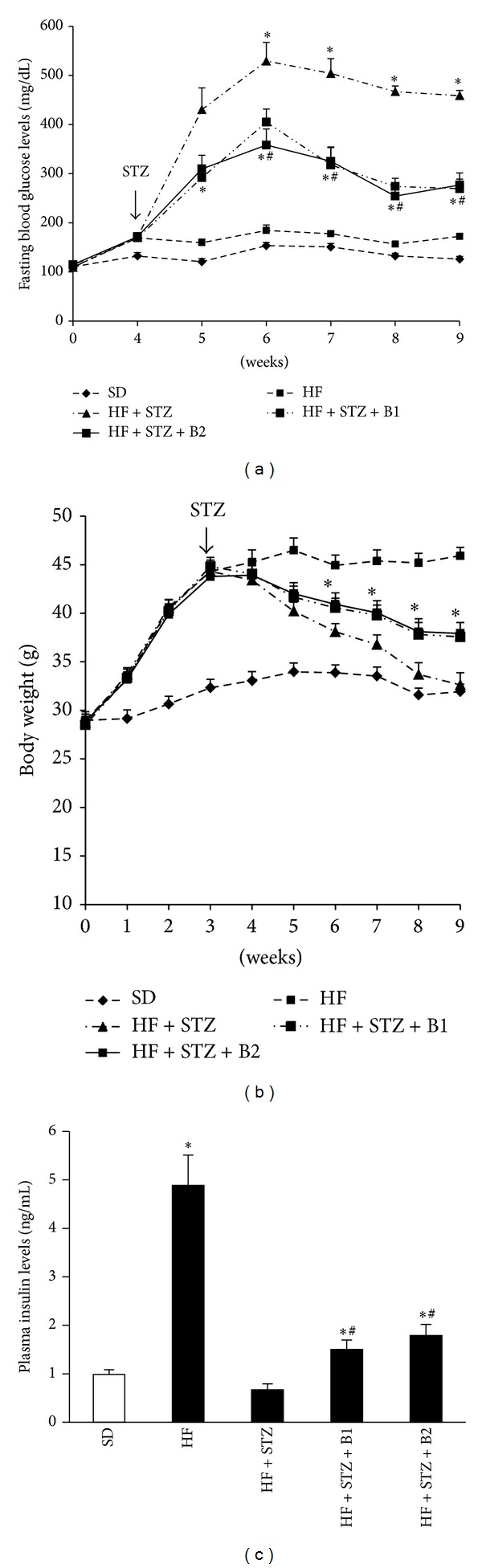
Dietary intake of baicalein ameliorated hyperglycemia in STZ-induced obese diabetic mice. MaleC57BL/6 mice (8 mo old) were fed a HF diet containing 0, 0.25, and 0.5 g baicalein/kg diet for 4 wks prior to administration of STZ (40 mg/kg for 3 days) and continued on the same diet for 5 wks. Aged-matched mice were fed a SD diet. Nonfasting blood glucose and (a) body weight (b) were monitored every wk after injection of STZ. Plasma insulin levels (c) in fasted mice were measured by ELISA. Data are means ± SE (*n* = 10 mice/group).  *P < 0.05 versus HF + STZ group,  ^#^ *P* < 0.05 versus SD group. SD: standard diet, HF: high-fat diet, B1: 0.25 g baicalein/kg diet, and B2: 0.5 g baicalein/kg diet.

We further assessed whether baicalein can prevent or ameliorate diabetes via promoting pancreatic *β*-cell function in a T2D mouse model that was generated through a combination of HF diet feeding and three consecutive injections of low-doses of STZ [30]. Regarding this, C57BL/6 mice (male, 8 months old) were fed the SD, a HF, or HF containing 0.25 g, 0.5 g, or 1.0 g/kg baicalein. Treatment with baicalein for 4 wk had no effect on body weight gain, fasting blood glucose levels, or food intake of mice fed the HF diet (data not shown), consistent with the observations from the animal study described above. After 4 wk of dietary treatment, STZ (40 mg/kg BW) was administrated (ip) for 3 consecutive days to induce mild to moderate diabetes mediated by a destruction of islet *β*-cells in mice [31]. Our data showed that baicalein significantly ameliorated STZ-induced hyperglycemia in diabetic mice fed 0.25 and 0.5 g/kg baicalein, with 0.5 g/kg baicalein producing the maximal protective effect (Figure 4(a)). Consistently, dietary ingestion of 0.25 and 0.5 g/kg baicalein ameliorated the loss of BW secondary to the development of diabetes [32] (Figure 4(b)), whereas food intake was not affected in diabetic mice (data not shown). In addition, insulin tolerance was not altered by baicalein treatment (data not shown), further confirming that this compound has no effect on insulin sensitivity. To determine if the improved glycemic control in mice that were fed the baicalein-supplemented diets is the result of improved islet function, we measured insulin levels in the plasma of control and baicalein-fed mice. As shown in Figure 4(c), plasma insulin levels in mice fed diets containing 0.25 and 0.5 g/kg baicalein were significantly greater as compared to those in nontreated diabetic mice, suggesting that baicalein may ameliorate hyperglycemia primarily through preserving islet *β*-cell function.

### 3.4. Dietary Intake of Baicalein Improved Islet *β*-Cell Mass in Obese Diabetic Mice

**Figure 5:**
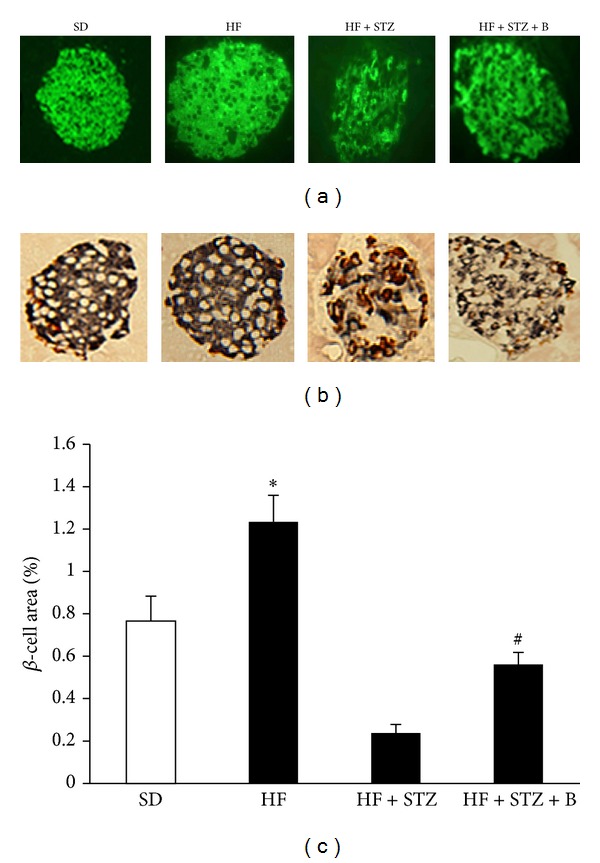
Baicalein supplementation improved pancreatic *β*-cell mass and reduced apoptosis. Pancreatic sections from mice given SD, HF, HF, and STZ administration (HF + STZ) and HF + STZ supplemented with baicalein (B, 0.5 g/kg diet) were fluorescently stained with insulin (a) or double stained with activated caspase-3 (red) and insulin (blue) (b). The *β*-cell mass was determined as described in the Methods section (c). Data are shown as means ± SE (*n* = 6–10 mice/group).  *P < 0.05 versus SD group,  ^#^ *P* < 0.05 versus HF + STZ group.

Since STZ causes diabetes by destroying islet *β*-cells [33], and the results from our first animal study showed that baicalein might improve *β*-cell function, we then examined whether baicalein treatment preserved *β*-cell mass in diabetic mice by using an immunohistochemical technique. We found that STZ administration severely decreased *β*-cell mass and insulin content and disrupted the islet architecture (Figures 5(a) and 5(b)). However, dietary provision of baicalein significantly improved islet *β*-cell mass and preserved its structure in diabetic mice (Figures 5(a) and 5(b)). Consistently, baicalein-treated diabetic mice displayed fewer apoptotic *β*-cells than untreated mice (Figure 5(c)), as determined by double immunolabeling of the activated caspase-3, a key protease involved in the terminal steps of cell apoptosis.

### 3.5. Baicalein Promoted Viability of Clonal *β*-Cells and Human Islets

**Figure 6:**
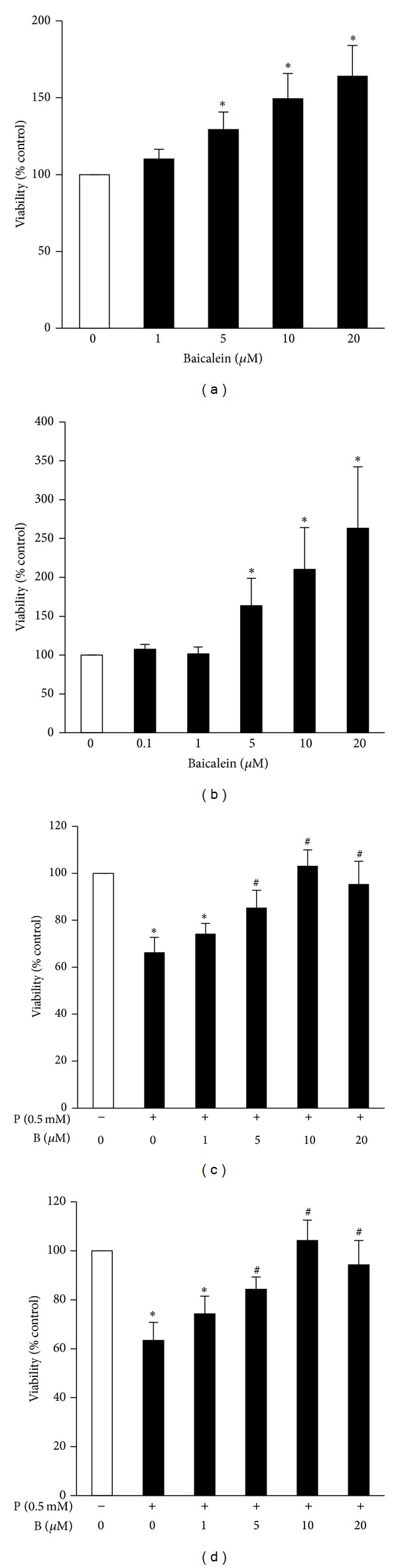
Baicalein promoted viability of *β*-cells and human islets. INS382/13 cells (a) or human islets (100 islets/well) (b) were cultured in RPMI medium containing 5.5 mM glucose and 2% FBS in the presence or absence of various concentrations of baicalein for 24 h. Cell viability was measured using a Cell Titer-Blue Cell viability assay kit. INS382/13 cells (c) or human islets (150 islets/well) (d) were cultured in RPMI medium containing 0.5 mM palmitate (P) or vehicle with or without various concentrations of baicalein (B) for 3 d. Cell viability was assessed as above. Data are expressed as mean ± SE from 3-4 independent experiments with quadruplicate determinations each.  *P < 0.05 versus control,  ^#^ *P* < 0.05 versus palmitate-alone treated cells or islets.

Our animal study showed that baicalein preserves functional *β*-cell mass. To investigate the mechanism underlying this protective action of baicalein, we assessed whether baicalein affects the viability of insulin-secreting cells and human islets. Our results showed that baicalein dose-dependently increased both INS382/13 cell (Figure 6(a)) and human islet (Figure 6(b)) viability, with 5–20 *μ*M exerting a protective action. To further determine whether baicalein is a survival factor for *β*-cells, we evaluated whether baicalein protects clonal *β*-cells and islets exposed to chronic hyperlipidemia. Incubation of INS382/13 cells (Figure 6(c)) or human islets (Figure 6(d)) with palmitate (0.5 mM) for 4 d significantly reduced cell viability. Addition of baicalein negated palmitate-induced reductions in cell viability, with 10 *μ*M baicalein, completely abolished this detrimental effect of palmitate on the cells, suggesting that this compound may be capable of protecting against hyperlipidemia-induced *β*-cell toxicity. It was reported that several flavonoids such as genistein can stimulate *β*-cell proliferation [27]. We considered the possibility that baicalein may promote *β*-cell survival by stimulating *β*-cell proliferation. However, our results showed that baicalein at 0.1 *μ*M to 20 *μ*M concentrations had no effect on *β*-cell proliferation as determined by a BrdU incorporation assay (data not shown).

### 3.6. Baicalein Promoted GSIS in Insulin-Secreting Cells and Human Islets

**Figure 7:**
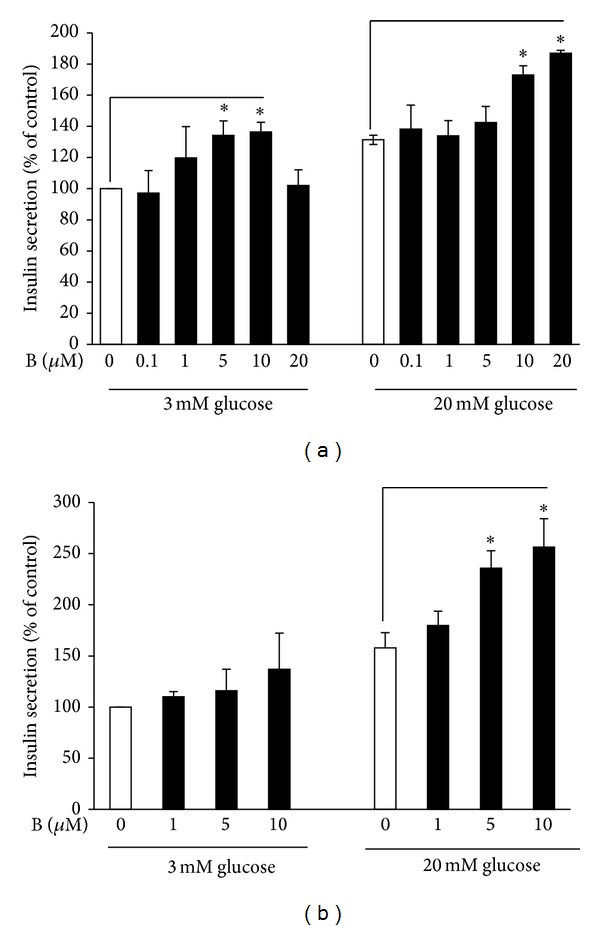
Baicalein enhanced glucose-stimulated insulin secretion from *β*-cells and human pancreatic islets. INS382/13 cells (a) or islets (b) were incubated in KRB buffer with various concentrations of baicalein (B) in the presence of 3 mM or 20 mM glucose at 37°C for 30 min. Insulin secreted into supernatants was measured by ELISA. Data are expressed as means ± SEM from 6 experiments for INS382/13 cells and 3 experiments for human isles in duplicate each.  *P < 0.05 versus control.

In the animal study, we found that baicalein may promote *β*-cell function and insulin secretion in response to glucose. To examine whether baicalein directly enhances GSIS in *β*-cells, we first cultured INS832/13 cells in basal (3 mM) or high (20 mM) glucose in the presence or absence of baicalein for 30 min. As shown in Figure 7(a), high glucose significantly induced insulin secretion and baicalein further dose-dependently augmented GSIS, with a maximal increase observed at a 10 *μ*M concentration (Figure 7(a)). We then tested whether baicalein has a similar effect on insulin secretion in pancreatic islets. As shown in Figure 7(b), baicalein had no effect on basal insulin secretion but significantly augmented GSIS in human islets, suggesting that these* in vitro* findings may have physiological relevance.

## 4. Discussion

Baicalein is a major active constituent in the medicinal herb* Scutellariae baicalensis Georgi*. Previous studies showed that it has potential beneficial effects on several chronic diseases. Recent studies showed that baicalin, the glucuronide form of baicalein, may exert beneficial effects on diabetes mellitus or diabetes-related complications [14–16]. However, whether and how baicalein exerts an antidiabetic effect is unclear. In the present study, we tested the antidiabetic potential of this natural compound using two mouse models, and we found that dietary intake of baicalein can improve glucose tolerance in middle-aged obese mice induced to become obese by HF diet feeding and ameliorate hyperglycemia in STZ-induced diabetic mice by preserving *β*-cell mass and protecting its function. While peripheral insulin resistance is common during obesity and aging in mice and humans, its progression to T2D is largely due to insulin secretory dysfunction and significant apoptosis of functional *β*-cells [5, 34–37], leading to an inability to compensate for insulin resistance. Indeed, those with T2D always manifest increased *β*-cell apoptosis and reduced *β*-cell mass [35, 36, 38]. In this context, it is tempting to speculate that baicalein may be a plant-derived novel antidiabetic compound by protecting *β*-cell mass and function, given that promotion or preservation of *β*-cell mass has been shown to be effective in preventing and treating diabetes [5, 34, 39–42].

In our animal studies, we used near middle-aged mice, because T2D usually occurs at middle and older ages in humans. We observed that baicalein treatment for 8 wks improved fasting blood glucose levels in obese mice. However, the mechanism by which baicalein treatment ameliorates fasting hyperglycemia is unclear, given that neither fasting plasma insulin levels nor peripheral insulin sensitivity was significantly modulated. A growing body of evidence indicates that obesity causes hepatic insulin resistance, leading to impaired regulation of glycogenolysis and gluconeogenesis by insulin, thereby increasing hepatic glucose production and output [43, 44]. Previous studies found that some of the flavonoids can reduce hepatic glucose production by suppressing the expression and enzymatic activities of phosphoenolpyruvate carboxykinase and glucose-6-phosphatase [45–48], two critical enzymes for gluconeogenesis in the liver. It is unknown whether flavonoid baicalein also has this effect, which is currently under investigation in this laboratory. In this study, we also found that baicalein treatment improved glucose tolerance in obese mice, which should primarily reflect a direct response of *β*-cells to circulating glucose, since whole body insulin sensitivity was not altered by baicalein treatment. Indeed, mice given baicalein displayed greater circulating insulin levels following ip injection of glucose compared to controls, suggesting that baicalein treatment may directly improve insulin secretion from pancreatic islets in response to a glucose challenge. We further explored whether baicalein directly protects pancreatic *β*-cell function by using a nongenetic mouse model of T2D, that was generated by employing a combination of feeding a HF diet and administering three mild doses (40 mg/kg) of STZ that does not cause diabetes in chow-fed mice, as demonstrated in our previous study [30]. This nongenetic diabetic mouse model manifests the metabolic characteristics of human T2D, including moderate levels of hyperglycemia, hyperlipidemia, insulin resistance, impaired insulin secretion, and reduced *β*-cell mass. The use of low-doses of STZ also minimizes the variability of diet-induced diabetes development and thus provides better experimental controls for evaluating the antidiabetic effects of this compound. The results in the present study show that baicalein protected against STZ-induced *β*-cell apoptosis, thereby leading to the improved *β*-cell mass in obese diabetic mice, which could be primarily attributable to its antidiabetic action, given that baicalein treatment did not alter other metabolic parameters such as insulin resistance, BW, or food intake.

Consistent with the animal data, we demonstrated that baicalein promoted insulin-secreting cell and pancreatic islet viability and insulin secretory function* in vitro*. Our results showing that 5 *μ*M baicalein can improve the viability of INS1 and human islet cells and stimulate insulin secretion might be of biological relevance, given that this dose of baicalein used in the present study may overlap with the physiologically achievable levels in the plasma following dietary intake. It was reported that baicalein can be absorbed from the stomach and the small intestine due to its high lipophilicity and low molecular weight [49, 50]. In fact, it was found that after oral administration of baicalein to rodents, the plasma concentrations of total baicalein peaked at 10 *μ*M and can be maintained at a 1 *μ*M concentration for 36 h [51, 52]. Collectively, these results obtained from both* in vivo* and* in vitro* experiments strongly suggest that baicalein may act directly on the pancreatic *β*-cell to enhance its function, thereby exerting an antidiabetic effect.

However, the exact mechanism underlying such a cytoprotective effect of this compound on *β*-cells is presently unknown. 12/15-Lipoxygenase (LO) is a member of the LO family that catalyzes conversion of arachidonic acid to 12-hydroperoxyeicosatetraenoic acid, which is subsequently reduced to 12-hydroxyeicosatetraenoic acid, an inflammatory mediator [53]. 12/15-LO is expressed in both rodent and human islets and its expression and activity are upregulated in HF diet-induced obese mice [54]. Recent studies have shown that 12-LO knockout mice are resistant to islet *β*-cell damage induced by HF feeding [54] or low-dose STZ administration [55], suggesting that this enzyme plays a crucial role in mediating metabolic stress-induced pancreatic *β*-cell damage. Baicalein has been shown to be a potent inhibitor of 12/15-LO [56]. Therefore, it is intriguing to speculate that baicalein treatment may prevent islet *β*-cells from HF feeding and STZ-induced damage, which is an ongoing investigation in this laboratory.

Our results show that baicalein could significantly stimulate insulin secretion in *β*-cell lines and human islets. However, the mechanism is unclear. It is well characterized that glucose induces insulin secretion through glycolysis and mitochondrial oxidation in *β*-cells. These events cause an increase in the intracellular ATP/ADP ratio, which sequentially leads to closure of K_ATP_ channels, depolarization of the cell membranes, Ca^2+^ influx, and ultimate exocytosis of insulin-containing granules [57]. Our* in vitro* study showed that baicalein treatment increases the viability of clonal *β*-cells and human islets. Increased cell viability could produce more nicotinamide adenine dinucleotide (NADH) and nicotinamide adenine dinucleotide phosphate (NADPH), leading to enhanced mitochondrial function to increase ATP production, which may in turn enhance insulin secretion in *β*-cells. Therefore, the effect of baicalein on insulin secretion may be mediated through improving mitochondrial metabolism, thereby increasing ATP generation in *β*-cells [58]. There is also the possibility that baicalein stimulates insulin secretion through a cAMP-mediated mechanism. Cyclic AMP signaling plays an important role in *β*-cell function including insulin secretion [59]. It was reported that genistein, a flavonoid from soybean, acutely stimulated insulin secretion in pancreatic *β*-cells via a cAMP-dependent protein kinase pathway [28]. Baicalein was shown to activate the cAMP/PKA cascade in intestinal epithelial cells [60]. However, it is unknown whether baicalein can activate the cAMP/PKA pathway in *β*-cells, leading to insulin secretion.

In summary, this is the first report to our knowledge that dietary supplementation of baicalein significantly ameliorated hyperglycemia and increased blood insulin levels, concomitant with improved functional islet mass, in obese diabetic mice. In addition, baicalein directly improved *β*-cell and human islet viability and insulin secretion, suggesting that this natural compound may have a novel antidiabetic action. Future study should be directed at elucidating the molecular mechanism by which baicalein stimulates insulin secretion and promotes *β*-cell survival.
